# Salt in the Wound: Assessing Pathogen Susceptibility in Amphibian Populations Across a Gradient of Salt Pollution

**DOI:** 10.1002/ece3.73598

**Published:** 2026-04-29

**Authors:** Rick A. Relyea, Jason T. Hoverman, Jessica Hua, Devin K. Jones, Melissa Lech, Brian Mattes, Candace Schermerhorn, Bryon Tuthill, Isabela Tuthill

**Affiliations:** ^1^ School of Natural Resources, Johnny Morris Institute University of Missouri Columbia Missouri USA; ^2^ Department of Forestry and Natural Resources Purdue University West Lafayette Indiana USA; ^3^ Forest and Wildlife Ecology Department University of Wisconsin Madison Wisconsin USA; ^4^ Department of Biological Sciences Rensselaer Polytechnic Institute Troy New York USA

**Keywords:** anthropogenic, disease ecology, evolution, pollution, species interactions

## Abstract

Freshwater salinization is an anthropogenic pollution issue that is receiving exponentially greater attention as we appreciate the global nature of the problem and the widespread lethal and sublethal impacts. A growing body of research is discovering that some freshwater taxa can evolve increased salt tolerance over generations, but such evolved tolerance often has tradeoffs in performance and fitness. We examined whether tadpoles collected from nine wood frog populations ( *Rana sylvatica* ), which have been exposed to generations of different elevated salt concentrations, differ in their susceptibility to common amphibian pathogens. When exposed to echinostomes, all tadpoles became infected, but there was no mortality and the populations had similar pathogen loads. When exposed to ranavirus (FV3), there were large population differences in survival, with populations from saltier ponds exhibiting lower survival. However, among the survivors, populations from saltier ponds had lower viral loads, suggesting higher resistance. Collectively, these results suggest that historic salt exposures in ponds can be associated with differences in susceptibility to some pathogens, although much more work needs to be conducted on additional populations, host species, and pathogen species.

## Graphical abstract

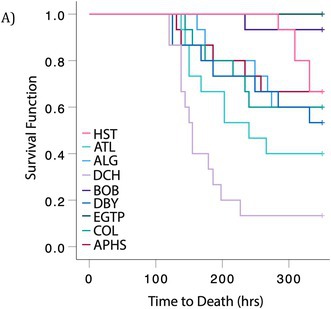

## 1 Introduction

Freshwater salinization is a rapidly growing issue around the world as increased amounts of salt (primarily NaCl) are entering into freshwater streams, rivers, wetlands, lakes, and groundwater. This salinization has a number of causes, including road‐salt applications in regions with snow and ice, mining, agricultural irrigation, and saltwater intrusion (Iglesias 2020; Szklarek et al. 2022). Given that freshwater taxa have lived and evolved under low‐salinity conditions for millions of years, they are generally poorly adapted to increased salt concentrations. Consequently, their performance is commonly impaired when exposed to salt, including altered behavior, physiology, growth, life history, and survival (Hintz and Relyea 2019; Arnott et al. 2020; Cunillera‐Montcusi et al. 2022).

Although anthropogenic salinization is relatively recent for freshwater taxa, there is some evidence for the evolution of increased salt tolerance. In zooplankton, for example, researchers have discovered that populations can evolve increased salt tolerance (Latta et al. 2012) and in some cases this can occur in just a few months (Coldsnow, Mattes, et al. 2017). However, these evolved populations experience a number of tradeoffs, including ablated circadian clocks and slower population growth (Coldsnow, Relyea, and Hurley 2017; Hintz et al. 2019). In amphibians, which have much longer generation times, evidence for evolved tolerance to salt is equivocal. In wood frogs, Buss et al. (2021) and Brady (2017) found no evidence of evolved tolerance in their wood frog populations. However, Relyea et al. (2024) reported that a population exposed to substantially higher salt concentrations than those examined in previous studies did exhibit elevated tolerance. This finding suggests that amphibians require exposure to salt concentrations beyond a threshold before tolerance can evolve. This suggests that evolved tolerance to salt is possible in amphibians. Interestingly, Relyea et al. (2024) did not observe any evolved tradeoffs in behavior, growth, or development. However, tradeoffs of evolved tolerance may be exhibited in other ways, including decreased resistance or tolerance to pathogens and parasites (hereafter the terms will be used interchangeably).

Pathogens (e.g., viruses, bacteria, helminths, fungi) are ubiquitous components of natural systems. Given the potentially devastating effects of infectious diseases, researchers have turned to disease ecology to better understand host‐pathogen interactions (Blaustein et al. 2012; Gervasi et al. 2013; Johnson et al. 2015). This is especially important given the growing influence of human activities on ecosystems (Corlett 2015). Anthropogenic changes to the environment have the potential to directly or indirectly alter host‐pathogen interactions. This is evident with chemical contaminants that frequently cause immunosuppression (Marcogliese and Pietrock 2011). For instance, research has demonstrated that chemical contaminants such as pesticides and per/polyfluoroalkyl substances (PFAS) can increase the risk of infection or accelerate disease progression (Rohr et al. 2008a, 2008b; Dietrich et al. 2014; Doublet et al. 2015; Brown et al. 2021; Lech et al. 2022). Although research examining the influence of salt on host‐pathogen interactions is limited, there is some evidence that salt exposure can lead to immunosuppressive effects in larval amphibians, which have the potential to influence disease dynamics through changes in lymphocytes (Milotic et al. 2017). Similarly, wood frog tadpoles exposed to NaCl salt had 170% more parasites encysted than individuals not exposed to NaCl salt (Buss and Hua 2018).

Wood frogs have emerged as a model system to explore the effects of freshwater salinization and pathogens. They are broadly distributed across northern North America where salt (primarily NaCl) is heavily used in the winter. Additionally, because they are one of the first amphibians to emerge in the spring, they can be exposed to salt via runoff during the spring thaw. Recently, several studies have demonstrated widespread variation in wood frog exposure to NaCl in their natural habitats and their tolerance to NaCl (Brady 2017; Relyea et al. 2024). Building on this work, we used wood frog populations from ponds with varying levels of salt concentrations to examine whether past exposure to salt was associated with an increased susceptibility to two common amphibian pathogens: the viral pathogen ranavirus (frog virus 3) and trematodes in the echinostome group (see below). If persistent exposure to salinization and evolved tolerance to salt leads to a tradeoff with immune function, we predicted that amphibian populations that are exposed to increased salt concentrations in their natal ponds would experience higher pathogen loads and increased pathogen‐induced mortality (Milotic et al. 2017).

### 1.1 Pathogen Background

Ranaviruses are widely distributed viral pathogens of ectothermic vertebrates and have been implicated in mortality events across the globe (Chinchar et al. 2009; Gray and Chinchar 2015). Frog virus 3 (FV3) is one strain of ranavirus that is broadly found in North America amphibian populations (Marschang et al. 2025). FV3 is transmitted via exposure to contaminated environments (water or soil), contact with infected individuals, and ingestion of infected tissue (i.e., predation, cannibalism, or necrophagy (Chinchar et al. 2009; Gray and Chinchar 2015)). Host mortality is caused by cell death in the liver, kidney, and spleen and signs of FV3 infection include lesions, swelling of the limbs or body, and hemorrhaging (Gray et al. 2009). Tadpoles and recently metamorphosed juveniles are more frequently reported in mortality events compared to adults (Brunner et al. 2015; Gray et al. 2009).

Echinostomes are digenetic trematodes that cycle through multiple hosts during their lifecycle. Their free‐swimming cercariae infect amphibians, targeting kidney tissue and forming metacercarial cysts (Billet et al. 2020; Toledo and Fried 2005). Signs of echinostome infection include edema and hemorrhaging (Johnson and McKenzie 2009). These infections can impair amphibian growth and development and can also lead to kidney failure and mortality (Hua et al. 2016; Orlofske et al. 2017; Strasburg and Boone 2021). The severity of these adverse effects depends on infection intensity, with more severe pathology occurring as the number of parasites increases (Holland et al. 2007), although the parasites may also be directly susceptible to higher salt concentrations.

## 2 Methods

### 2.1 Animal Collection

The animals used in the pathogen‐exposure experiments were part of a larger effort to examine the salt tolerance among amphibian populations that differed in natural exposure to salt‐polluted water bodies (Relyea et al. 2023, 2024; see map in Figure S1). We sampled the salt concentrations in the full set of ponds in 2022 and a subset of the ponds in 2024; the pattern of low‐ to high‐salt concentrations remained consistent. We used nine populations of wood frogs that we collected as newly oviposited egg masses from local ponds and wetlands in Rensselaer County, NY (USA). Egg collections occurred during 6–12 April 2022 and we collected 10 egg masses from most populations (only 7 masses were available in the EGTP population) to provide a good genetic representation of each population. At the same time, we measured chloride concentrations in the ponds and wetlands. The eggs were hatched in outdoor wading pools containing aged well water, which had low chloride levels (18 mg Cl−/L); all eggs hatched within a 2‐d period (14–15 April). Hatchlings were fed high‐protein rabbit pellets (i.e., ground alfalfa; Blue Seal Fresh Show Hutch Deluxe). We first used tadpoles from the nine populations to quantify population‐level variation in salt tolerance, salt‐induced tolerance, growth, behavior, and development (Relyea et al. 2023, 2024) and then used individuals from the same clutches to assess pathogen susceptibility. The egg collection was conducted under a state collecting permit and the experiments were part of an approved IACUC protocol (REL‐001‐22).

**FIGURE 1:**
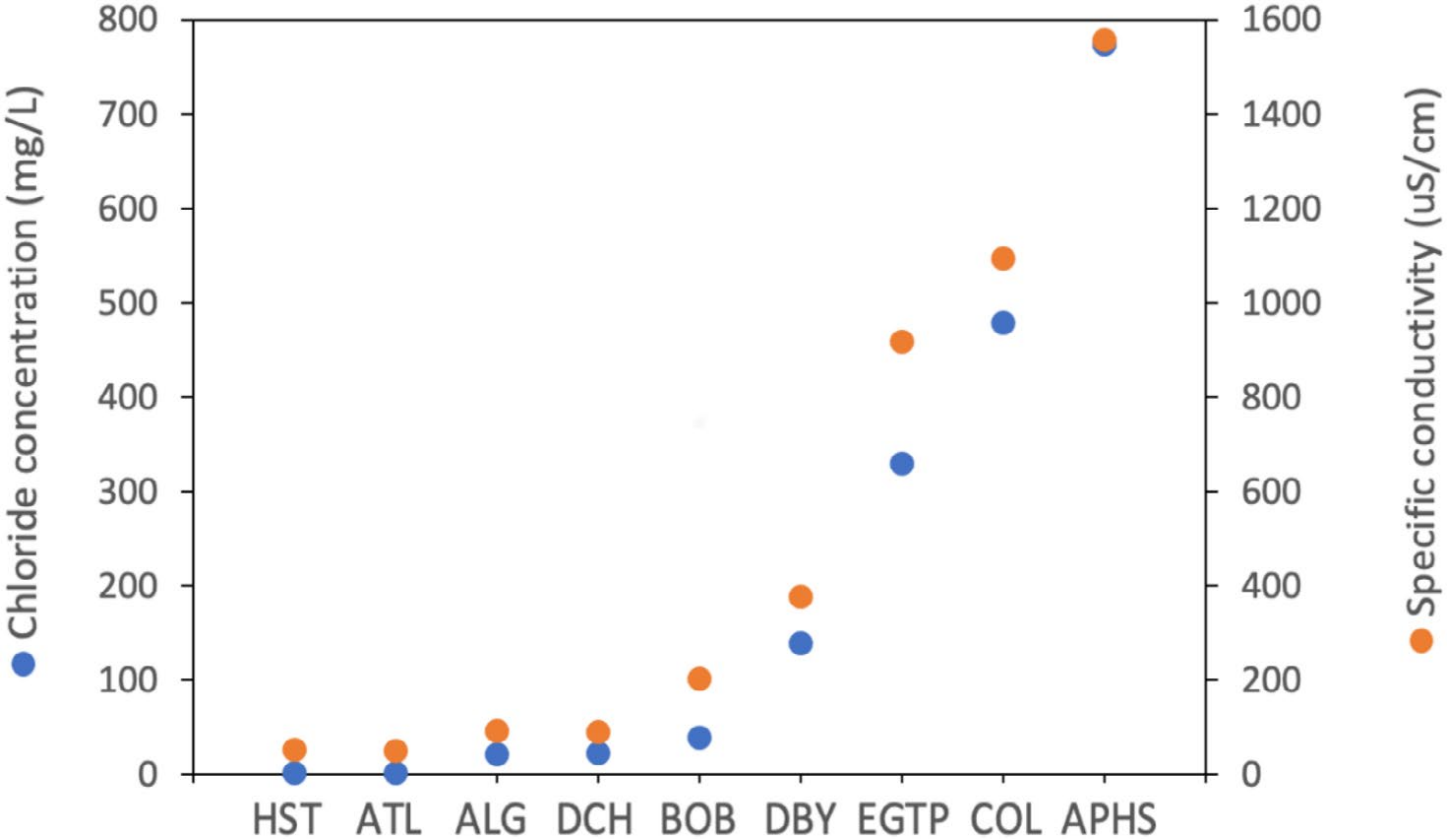
The range of salt concentrations found in the nine ponds where the wood frogs were collected. Reprinted from Relyea et al. (2023).

While the pristine condition for freshwater is typically < 10 mg Cl^−^/L, we discovered a wide range of salt concentrations in these water bodies, including one that approached 800 mg Cl^−^/L (Figure 1). Once we discovered population‐level differences in salt tolerance and induced tolerance, we decided to examine whether the same clutches of tadpoles from these populations also differed in their susceptibility to two common amphibian pathogens: FV3 and trematodes. For both experiments, tadpoles were raised in a lab at 20°C, with a 15:9 h day/night cycle. All water was filtered and UV‐irradiated (temperature = 18°C, salinity = 24 mg Cl^−^/L, pH = 7.1 to 7.6). Initial mass and Gosner stage for each population in each experiment are detailed in Table 1.

**TABLE 1:** Initial mass (mg) and developmental stage (Gosner 1960) of nine larval wood frog populations in the two pathogen experiments.

A. Population	FV3 experiment		Trematode experiment	
	Mass	Stage	Mass	Stage
ALG	101 ± 9	26.5 ± 0.2	115 ± 14	29.2 ± 1.0
APHS	120 ± 3	27.2 ± 0.1	116 ± 6	29.0 ± 0.3
ATL	117 ± 8	27.2 ± 0.1	132 ± 18	30.2 ± 1.2
BOB	124 ± 7	27.4 ± 0.2	105 ± 11	29.6 ± 1.0
COL	140 ± 6	27.6 ± 0.2	145 ± 13	31.6 ± 0.6
DBY	112 ± 5	27.3 ± 0.2	153 ± 14	31.6 ± 1.2
DCH	128 ± 9	27.7 ± 0.2	149 ± 10	31.6 ± 0.6
EGTP	125 ± 7	27.3 ± 0.2	195 ± 13	32.2 ± 0.4
HST	143 ± 8	27.8 ± 0.2	177 ± 28	32.0 ± 0.7

When rearing populations from natal ponds of different salt concentrations, one could test for differences in pathogen susceptibility by testing all populations under a low‐salt concentration, testing all populations under a high‐salt concentration, or testing each population under the unique salt concentrations of each pond. We chose the first option since some populations would not survive a high‐salt concentration and using unique salt concentrations of each pond for each population would confound the wood frog population being tested for pathogen susceptibility with the salt concentration of that pond.

### 2.2 FV3 Experiment

#### 2.2.1 FV3 Culturing

To generate FV3, we passaged FV3 Isolate, WP4‐2C, (Brunner Lab Washington State University, WA, USA) through fathead minnow (FHM) CCL‐42 cells (ATCC, Manassas, VA, USA). Fathead minnow cells were grown in Dulbecco's Modified Essential Medium (DMEM) with Hank's balanced salts (HyClone, Logan, UT, USA, Cat #:SH30003.03), pH 7.4, supplemented with 10% Fetal Bovine Serum (FBS) at 25°C and atmospheric CO_2_ conditions. Prior to FV3 culture, FHM cells were sub‐cultured in 175 cm^2^ flasks and allowed to grow to 75% confluency. Flasks were then inoculated with 50 μL of stock viral suspension tittered at 5.55 × 10^7^ PFU/mL and allowed to grow at 20°C until 75% of FHM cells showed signs of cytopathic effect. Cells were subsequently sub‐cultured into new 75% confluent flasks three successive times.

The resulting culture was collected, centrifuged at 200*rcf, and filtered through a 0.45‐μm polyethersulfone membrane filter. Filtrate was tittered via plaque assay using 2% methyl cellulose‐viscous culture media (2% MC‐VCM) without antibiotics (2% Methyl cellulose, DMEM, 5% FBS, pH 7.4). Serial dilutions from 1:10 to 1:1,000,000 of FV3 preparation were used to assess viral concentration in 25‐cm^2^ flasks with 2% MC‐VCM at 20°C. Cells were allowed to incubate for 9 days, at which time flasks were fixed and stained with 10% formalin/EtOH (v/v) with 0.1% Crystal Violet (w/v). Flasks were visualized under a microscope (Olympus Model CKX53SF, Tokyo, Japan) for plaque formation and counted. The final titer was assessed at 6.66 × 10^6^ PFU/mL. Viral samples were stored at −80°C until the start of the experiment.

#### 2.2.2 FV3 Exposure

On 9 May, we exposed tadpoles from nine wood frog populations to a control or FV3 treatment (1 × 10^3^ PFU/mL). We replicated each control treatment 5 times and each FV3 treatment 15 times for a total of 180 experimental units. Experimental units were initially 50‐mL plastic cups containing 40 mL of water and a single tadpole. For all experimental units, we added 13.3 μL of virus stock (6.66 × 10^6^ PFU/mL) to the cups to generate a final viral concentration of 1 × 10^3^ PFU/mL. For all control experimental units, we added 13.3 μL of virus‐free media (DMEM). These ranavirus exposure doses generate infections that are comparable to field‐collected amphibians (Brunner et al. 2015, 2025; Hoverman et al. 2011, 2012).

After 2 days of exposure, all tadpoles and their 40 mL of water were transferred into 1‐L containers containing 460 mL of filtered water, bringing the total water volume up to 500 mL. We fed the tadpoles ground TetraMin fish food, *ad libitum*, for 2 weeks. On 17 May (Day 8), we conducted a partial water change by removing 400 mL of water and replacing it with new filtered water. Survival checks were conducted approximately 2 to 6 times each day, with less frequent checks as death rates slowed over time.

On 25 May (Day 16), we euthanized and preserved all surviving individuals in liquid nitrogen. Tadpoles that died during the experiment were placed into microcentrifuge tubes that were pre‐filled with 95% EtOH and placed in the freezer at −20°C.

#### 2.2.3 Quantifying FV3 Loads

We determined FV3 loads in livers using quantitative polymerase chain reaction (qPCR). To isolate viral DNA, we used Qiagen DNEasy Blood and Tissue kit (Qiagen, Germany, Cat #: 69506). The DNA was then quantified by analyzing 1 μL of DNA using NanoDrop One (Thermo Fisher Scientific, Madison, WI). We then performed qPCR using a Bio‐Rad CFX Duet (Bio‐Rad, Hercules, CA, USA). Each PCR reaction consisted of 1 μL Custom TaqMan Gene Expression Assays (Applied Biosystems, Thermo Fisher Scientific, Warrington, UK, Cat # 4331348) for FV3, 10 μL 2× TaqMan (Applied Biosystems, Thermo Fisher Scientific, Warrington, UK, Cat # 4304437), and 1 μL sample DNA or standard. We used a synthetic double‐stranded DNA gBlock Standard (IDT Technologies, Coralville, IA, USA) for the FV3 major capsid protein (Stilwell et al. 2018). Serial dilutions of standards ranging from 1.55 × 10^1^ to 1.55 × 10^9^ copies*μL^−1^ were used to confirm absolute quantities of pathogen DNA. All controls, standards, and unknowns were run in duplicate. Primers used are listed in the Supplement. We considered all samples that peaked prior to cycle 35 to be positive (Miller et al. 2015). Using the starting quantity (SQ) generated by the qPCR corresponding to pathogen DNA particles, sample volume, and ng DNA detected via NanoDrop, pathogen load was calculated (ng/mL).

### 2.3 Trematode Experiment

#### 2.3.1 Trematode Collection

To obtain trematodes, we collected ramshorn snails ( *Helisoma trivolvis* ) from a permanent pond at the Purdue Wildlife Area in West Lafayette, IN (USA) from 23 to 26 May 2022 using dipnets. Snails were screened for echinostome infection following methods described in Szuroczki and Richardson (2009). Briefly, snails were individually placed in 50‐mL Falcon centrifuge tubes filled with 35 mL of aged well water (filtered and UV‐irradiated) and placed under a light for 1 h to induce trematode shedding. Infected snails were housed together in 15‐L tubs filled with 10 L of aged well water until they were shipped overnight to Rensselaer Polytechnic Institute (RPI) on 31 May 2022. For shipment, the snails were placed in 500‐mL Nalgene bottles filled with aged well water. After arriving at RPI, the infected snails were placed in 10‐L tubs filled with 8 L of aged well water until used as a source of shed trematodes for the experiment. During this time, the snails were not fed.

#### 2.3.2 Trematode Exposure

The trematode‐exposure experiment was started on 3 June 2022. For the control treatment, we used 10 tadpoles from each population (total = 90) with each placed individually in 50‐mL cups, each containing 30 mL of water. For the parasite treatment, we used 25 tadpoles from each population (total = 225) also placed individually in 50‐mL cups. For the parasite exposures, our target number was 100 cercariae for each tadpole, which required that we shed trematodes from the snails twice, producing two sequential exposures that took place approximately 1 h apart. We shed trematodes from the snails following the method described above. We then pooled the cercariae from all snails and took a 1‐mL aliquot to determine cercarial density in the samples. For the first exposure, the cercarial density was 12 per mL, so we added 5 mL of pooled cercariae to each experimental unit for a total of ~60 cercariae. For the second exposure, the mean cercarial density was 20 per mL, so we added an additional 2 mL of pooled cercariae containing ~40 cercariae. Control tadpoles were given an equivalent amount of clean water for each exposure (total water volume per cup = 37 mL). These trematode exposure doses generate infections that are comparable to field‐collected amphibians (Johnson and Hoverman 2012; Hoverman et al. 2013). Tadpoles were not fed rabbit chow (i.e., ground alfalfa) while being held.

Tadpoles were exposed to trematodes for 6 h (following the second dosing) and then all tadpoles (including controls) were transferred into clean water for the duration of the experiment. Three days after trematode exposure, tadpoles were euthanized with an overdose of MS‐222 and preserved in 10% buffered formalin. We chose 3 days for terminating the experiment to focus on initial infection success of the parasite and limit confounding effects of possible parasite clearance (Johnson et al. 2011). Preserved tadpoles were shipped to Purdue University for parasite quantification and morphometric measurements. Prior to dissection, we recorded mass, snout‐vent length (SVL), and developmental stage (Gosner 1960) for each individual.

#### 2.3.3 Quantifying Trematode Loads

To quantify parasite load, we removed the kidneys, pressed them between two glass slides, and examined them for cysts using an inverted compound microscope. We counted the cysts twice to ensure accuracy. Control tadpoles were also dissected, which allowed us to confirm there were no infections prior to parasite exposures.

### 2.4 Statistical Analyses

For the FV3 experiment, we examined tadpole survival and viral load among the nine populations. We began by comparing the cumulative survival probabilities of the populations using a Kaplan Meier (Generalized Wilcoxon) test and then examined whether the differences in averaged cumulative survival probabilities were correlated with the saltiness of the natal ponds (using Kendall's Tau non‐parametric correlation analysis). The averaged cumulative survival probability is a proxy metric to compare susceptibility to FV3 among populations. Next, we examined population differences in infection prevalence using a generalized linear model with a binomial logit link function and then examined whether the differences in infection prevalence were correlated with the saltiness of the natal ponds (using Kendall's Tau non‐parametric correlation analysis). Lastly, we examined whether the populations differed in viral load by examining viral load in both the dead tadpoles and the surviving tadpoles using analysis of variance. Assumptions for equal variances were not met for viral loads of dead tadpoles, so we conducted non‐parametric analyses by first ranked transforming these data. With these insights, we then examined whether viral loads for the populations were correlated with the saltiness of the natal ponds (using Kendall's Tau non‐parametric correlation analysis). For all FV3 correlation analyses, we used a one‐sided test because we had a priori expectations for the relationships tested.

For the trematode experiment, we did not analyze survival since it was 100% for control and exposed tadpoles. For trematode load (number of cysts log transformed), the dataset failed tests of normality and homoskedasticity for analysis of variance. Thus, we conducted a nonparametric Kruskal–Wallis test to examine population differences (fixed effect) in trematode load (group rank sums). We also conducted a linear regression to examine whether the salt concentrations (log transformed) of the nine natal ponds predicted trematode load (log transformed). Analyses were conducted in the R statistical environment v4.5.2 (R Development Core Team 2025) using the base or *car* packages (Fox and Weisberg 2019). For all statistical tests, we defined significant as *a* < 0.05 and marginally significant as 0.05 < *a* < 0.1.

## 3 Results

### 3.1 FV3 Experiment

In the FV3 experiment, five of the 45 control tadpoles (11%) were categorized as being positive for an infection (spread across 3 populations); across all populations the survival in the control was 98% (i.e., eight populations experienced 100% survival, one population had a single death for 80% survival). The mean (±SE) viral load across all populations in the control treatments was very low (0.4 ± 0.2 copies/ng).

**FIGURE 2:**
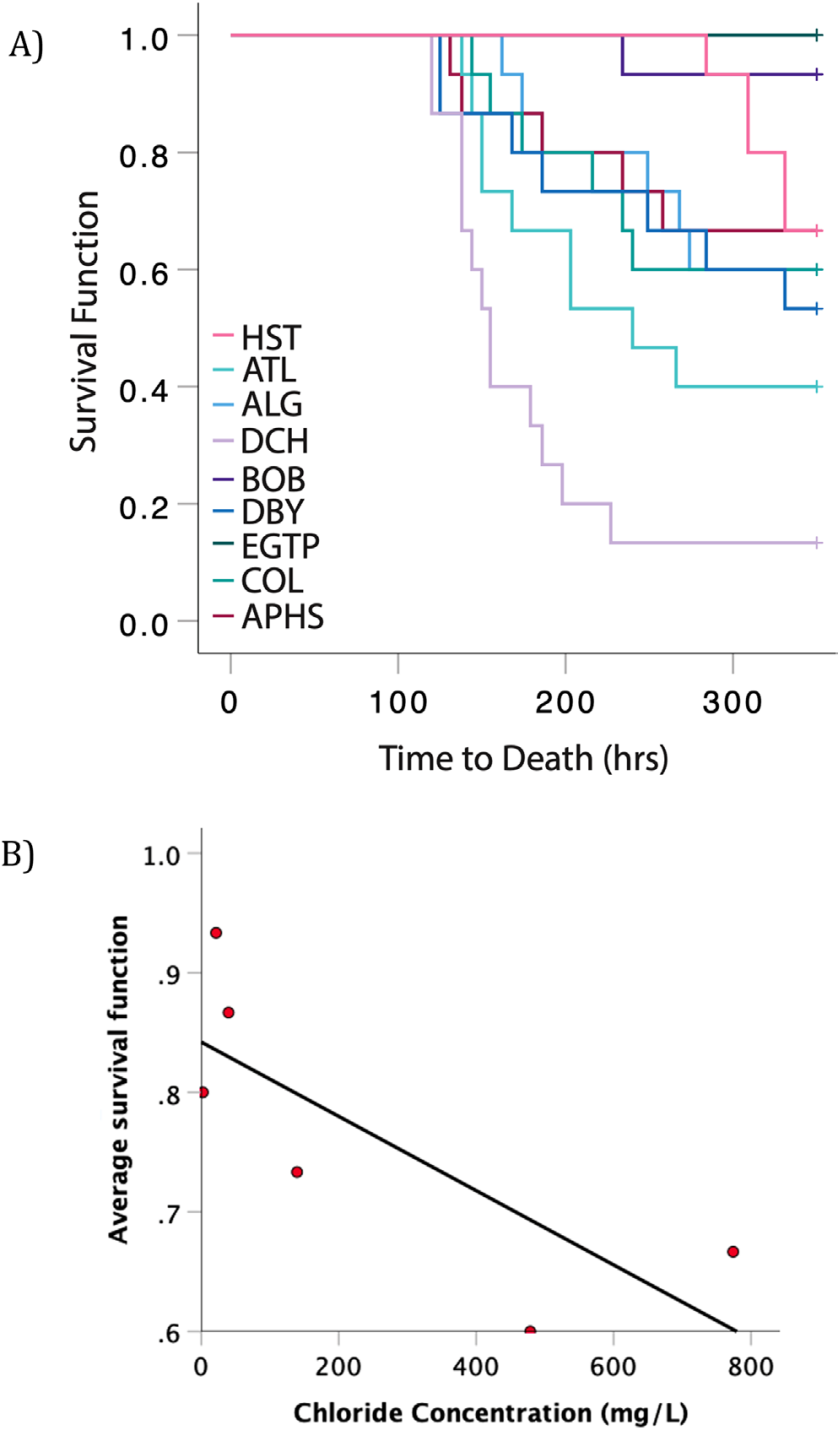
(A) The survival function of nine larval wood frog populations and their viral loads when exposed to FV3. The populations were collected as eggs from a range of salt‐polluted ponds. Viral load data are means ±1 SE. The populations are ordered in terms of the saltiness of their natal ponds (see Figure 1). (B) The correlation between the average cumulative survival probabilities of the populations exposed to FV3 and the saltiness of the natal ponds where the wood frog eggs were collected. Note that there are no data for the EGTP population, which experienced no mortality. Only six data points are visible as a result of some data points having values that overlap.

Among the nine FV3‐exposed populations, we found significant differences in tadpole survival probabilities, with survival ranging from 13 to 100% (*χ* ^2^ = 58.5, *p* < 0.001; Figure 2A). We also found a marginally significant correlation of higher survival functions in populations from natal ponds containing lower salt concentrations (*p* = 0.053; *R* ^2^ = −0.47, Figure 2B).

**FIGURE 3:**
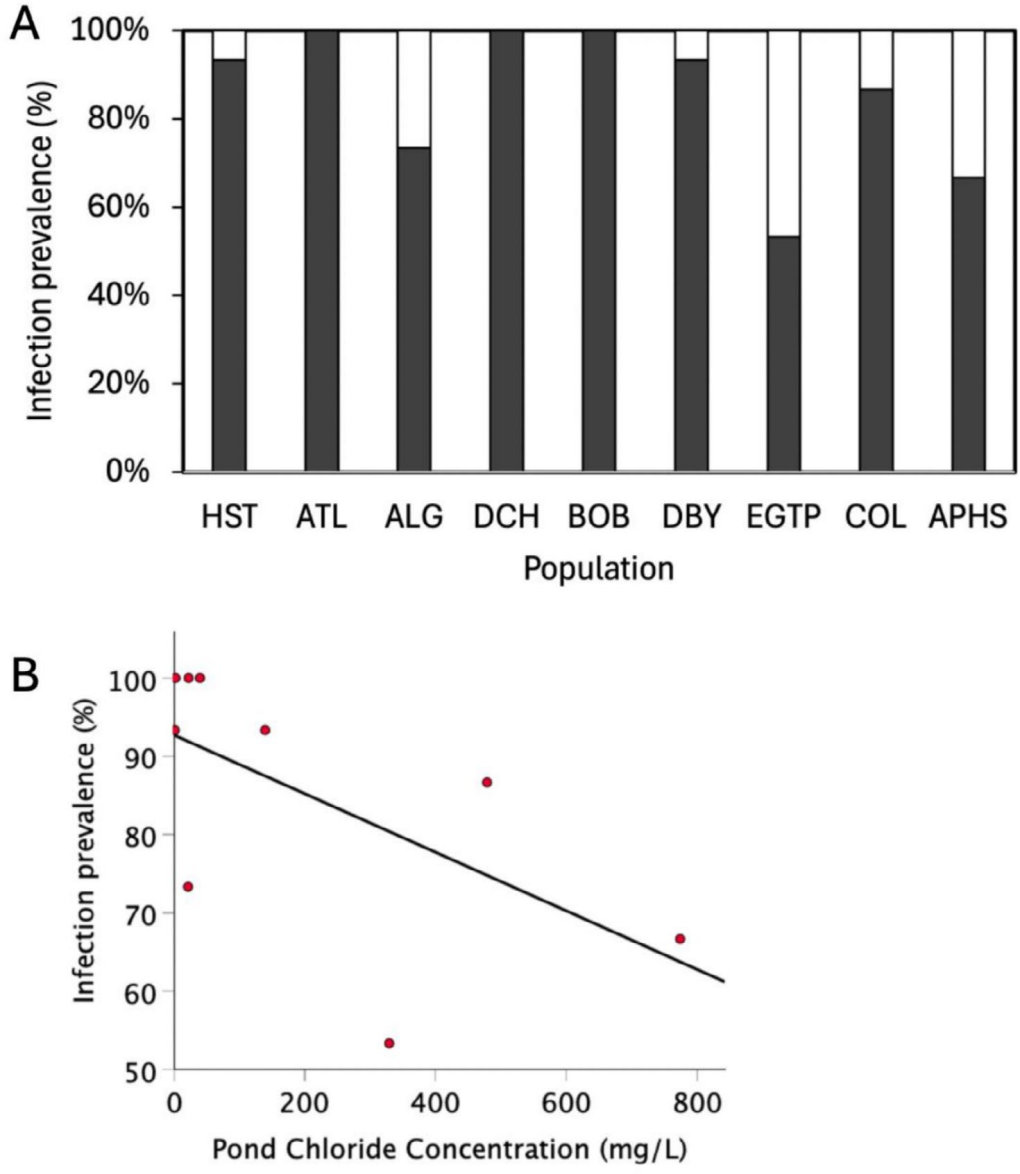
Ranavirus infection prevalence in nine populations of wood frog tadpoles exposed to FV3. (A) Infection prevalence. (B) There was a marginally significant, negative correlation between infection prevalence and the saltiness of the natal ponds.

In regard to infection prevalence, we found that the nine populations exhibited differences in the percent of individuals that were infected by ranavirus (*χ* ^2^ = 29.6, *p* < 0.001; Figure 3A). When we correlated infection prevalence against the saltiness of the natal ponds, we found a marginally significant, negative correlation (*p* = 0.067; *R* ^2^ = 0.1; Figure 2B). Thus, we observed a trend of amphibians from saltier ponds experiencing reduced ranavirus prevalence.

**FIGURE 4:**
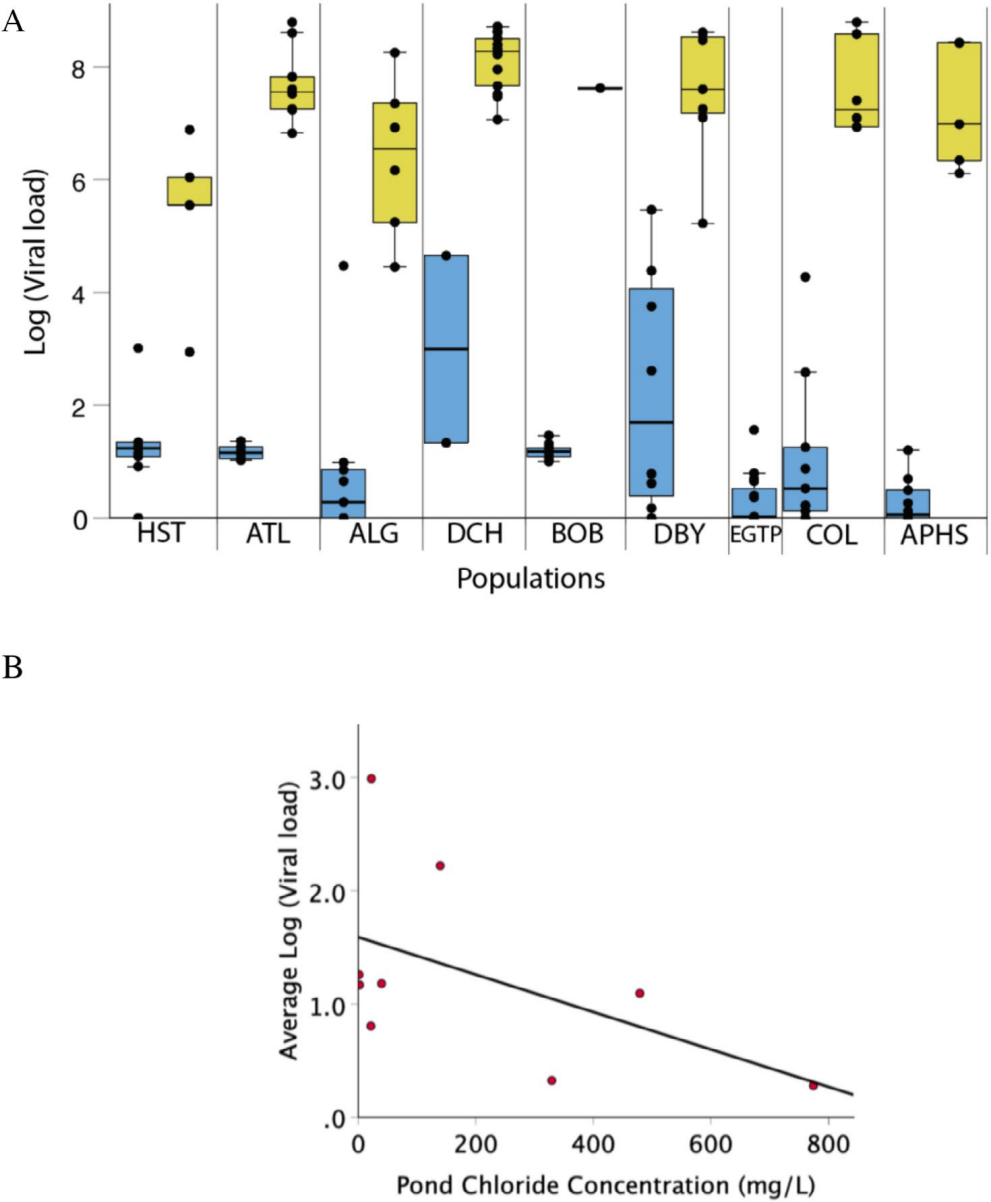
Viral loads in nine populations of wood frog tadpoles exposed to FV3. Viral loads were examined for (A) dead tadpoles (yellow bars) and surviving tadpoles (blue bars). (B) For the surviving tadpoles, there was a marginally significant, negative correlation between log(viral load) and the saltiness of the natal ponds.

In our examination of the viral loads, we detected population differences among dead tadpoles (F_7,44_ = 5.0, *p* < 0.001; Figure 4A, yellow bars) and among surviving tadpoles (F_8,74_ = 6.4, *p* < 0.001; Figure 4A, blue bars). When we compared the viral loads against the saltiness of the nine natal ponds, we found no correlation for the dead tadpoles (*p* = 0.45, *R* ^2^ = −0.22), but there was a marginal negative correlation for the surviving tadpoles (*p* = 0.072, *R* ^2^ = −0.39; Figure 4B). While this analysis was based on population means, a similar analysis using the individuals (raised independently) detected the same negative correlation, but was more significant as a result of the increased statistical power (*p* = 0.003; *R* ^2^ = −0.29). This means that surviving tadpoles from the saltiest ponds had the lowest viral loads. We also noted that the pathogen loads in the dead tadpoles were approximately 10^6^ times higher than the surviving tadpoles.

### 3.2 Trematode Experiment

**FIGURE 5:**
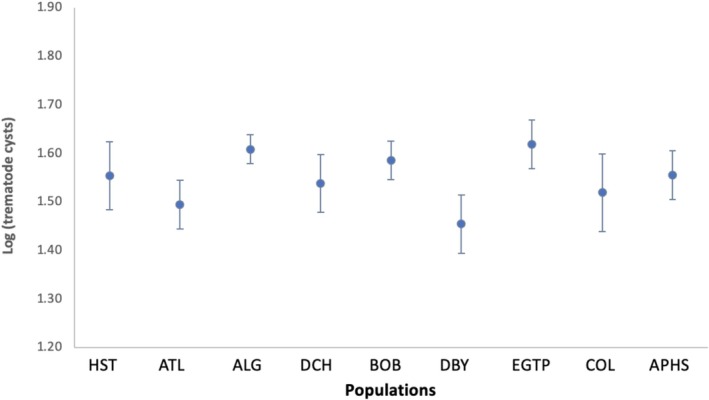
The abundance of trematode cysts among nine populations of wood frog tadpoles that were collected as eggs from a range of salt‐polluted water bodies and then experimentally exposed to trematodes (log‐transformed data are means ±1 SE). The populations are ordered in terms of the saltiness of their natal ponds (see Figure 1).

In the trematode experiment, tadpole survival was 100% in the control and trematode‐exposure treatments. While control tadpoles had no trematode infections, every tadpole exposed to trematodes experienced an infection, ranging from 5 to 125 cysts per individual. However, there were no signs of disease (e.g., edema, hemorrhages) observed following parasite exposure. Additionally, there were no differences in the number of cysts among the nine populations (*χ* ^2^ = 7.054, df = 8, *p* = 0.531; Figure 5). Additionally, the salinity of the nine natal ponds was not predictive of trematode load (F_1,222_ = 0.061 *p* = 0.805).

## 4 Discussion

The evolution of adaptations to local environments, including evolved tolerance to contaminants, is pervasive and often comes with performance and fitness tradeoffs (Bono et al. 2017). In this study, we tested for tradeoffs in pathogen susceptibility among amphibian populations from natal ponds spanning a wide range of salt concentrations, from 1 to 774 mg Cl^−^/L, which is well above the pristine condition of < 10 mg Cl^−^/L. Across these ponds, the population experiencing the highest salt concentration has much higher salt tolerance (Relyea et al. 2024). While we found no evidence that populations from saltier ponds have greater susceptibility to trematodes, we did find weak evidence that mortality rates following exposure to ranaviruses were higher for populations from saltier ponds. While we suspect these are evolved differences, we cannot rule out the possibility of maternal effects or long‐term impacts of the different salt environments when the eggs were first laid.

When tadpoles were exposed to echinostomes, we found no significant differences among the nine wood frog populations in survival or parasite load. This suggests that historic exposures to salt in wood frogs cause no change in susceptibility to echinostomes. This result is particularly interesting given that prior research on 15 wood frog populations in Pennsylvania found that echinostome load was negatively correlated to the evolved tolerance to a common insecticide and each pond's distance to agriculture (Hua et al. 2017). However, a subsequent study of eight of those Pennsylvania populations found the opposite trend (populations closer to agricultural fields had higher echinostome loads; Billet et al. 2021). At this early stage in research, all we can say is that the underlying mechanisms involved in evolved tolerance to salinization versus pesticides are likely different in ways that cause different patterns of parasite susceptibility. We also note that the ponds in our study were not surveyed for the presence of snails, the first intermediate hosts of echinostomes. If snails are rare in these ponds or if the parasites are susceptible to high salt concentrations, it is possible that a lack of evolutionary interactions between the host and parasite could have limited our ability to detect meaningful associations.

When tadpoles were exposed to the ranavirus FV3, we found weak population differences in survival, with higher survival in wood frog populations living in low‐salt ponds. This suggests that there may be a tradeoff between historic exposures to salt pollution and the ability to survive against FV3. This is consistent with the recent work of Hall et al. (2020), who found that wood frogs living in high‐salt ponds were more likely to experience die‐offs from ranavirus and that salt exposures caused higher odds of mortality when exposed to ranavirus in laboratory experiments. Although identifying the mechanism underlying this result was beyond the scope of the study, it is likely the consequence of a series of effects initiated by physiological changes associated with water balance. For amphibians, physiological functions associated with osmoregulation are likely upregulated in saline environments. In addition to the energy required to maintain water balance, stress hormones such as corticosterone are likely produced. Chronically elevated corticosterone levels are known to suppress immune function in amphibians. Thus, it is possible that the evolution of salt tolerance is associated with increased disease risk for amphibians that are challenged with highly virulent pathogens such as ranaviruses. Interestingly, the pathogen load of FV3 in the surviving tadpoles exhibited an opposite pattern, with survivors of populations from saltier ponds having marginally lower pathogen loads. This suggests that while individuals evolving in high‐salt ponds are more susceptible to FV3, those individuals that do not die exhibit some level of resistance to being infected, an ability to tolerate infections, or they are able to better clear the FV3 from their bodies.

In other studies of wood frog populations that vary in tolerance to different anthropogenic stressors (i.e., insecticides), researchers have also found differences in infection prevalence (i.e., percent infected). For example, Billet et al. (2021) found that wood frog populations living closer to agricultural fields (i.e., a proxy of historic pesticide exposure) were more susceptible to trematode infection (i.e., high parasite loads) but not more susceptible to ranavirus infection. In contrast, Hua et al. (2017) found that wood frog populations living closer to agricultural fields were less susceptible to trematode infections but more susceptible to ranavirus. Thus, the pattern between past exposures to different anthropogenic chemicals and susceptibility to amphibian pathogens is currently equivocal. Additional research could benefit from examining additional chemical pollutants in natural ponds, additional pathogens, and longer‐term exposure experiments.

## 5 Conclusions

This study demonstrates that anthropogenic freshwater salinization can influence disease dynamics in amphibians, revealing potential trade‐offs between evolved salt tolerance and pathogen susceptibility. While we found no evidence that salt history affects susceptibility to echinostomes, exposure to ranavirus (FV3) produced population‐level differences in susceptibility that were related to salt exposure. These contrasting outcomes underscore the complexity of host‐pathogen interactions under environmental change. The mechanisms underlying these patterns remain uncertain but may involve physiological stress, altered osmoregulation, or immunosuppression linked to chronic salt exposure. Future research should explore these pathways and expand to additional pathogens and amphibian species to determine whether similar trade‐offs occur more broadly. Our findings highlight that environmental stressors can have indirect effects beyond toxicity, influencing disease outcomes and potentially shaping population persistence. As freshwater salinization continues to rise globally, integrating evolutionary and disease ecology will be critical for predicting wildlife responses and informing conservation strategies in a rapidly changing world.

## Author Contributions

**Rick A. Relyea:** project administration (equal), conceptualization (equal), methodology (equal), data curation (equal), formal analysis (equal); funding acquisition (equal). **Jason T. Hoverman:** project administration (equal) conceptulaization (equal), methodology (equal), formal analysis (equal). **Jessica Hua:** project administration (equal), conceptualization (equal), methodology (equal), formal analysis (equal), funding acquisition (equal). **Devin K. Jones:** conceptualization (equal), investigation (equal). **Melissa Lech:** conceptualization (equal), investigation (equal). **Brian Mattes:** investigation (equal). **Candace Schermerhorn:** investigation (equal). **Bryon Tuthill:** conceptualization (equal), investigation (equal). **Isabela Tuthill:** conceptualization (equal), investigation (equal).

## Funding

This work was supported by U.S. Department of Agriculture (10.13039/100000199), 1023388, 7005498. National Science Foundation (10.13039/100000001), 2137424, 2314625.

## Conflicts of Interest

The authors declare no conflicts of interest.

## Supporting information


**Figure S1:** Map of amphibian populations.

## Data Availability

All data and code have been uploaded as a supplement. All data will ultimately be publicly archived at EDI upon acceptance for publication.
