# Supplementary material for: Salt in the Wound: Assessing Pathogen Susceptibility in Amphibian Populations Across a Gradient of Salt Pollution
Source: Ecol Evol. 2026 Apr 29;16(5):e73598. doi: 10.1002/ece3.73598 (PMC13128336; doi:10.1002/ece3.73598)

Supplemental information

**Primers used to quantify FVS loads:**

FV3 Forward: 5’-ACACCACCGCCCAAAAGTAC-3’

FV3 Reverse: 5’-CCTCATCGTTCTGGCCATCAACCAC-3’

FV3 Probe: 5’-FAM-CATTATCCGCATCATGAACGG-ZEN-3’

**Figure S1. Map of amphibian populations.**


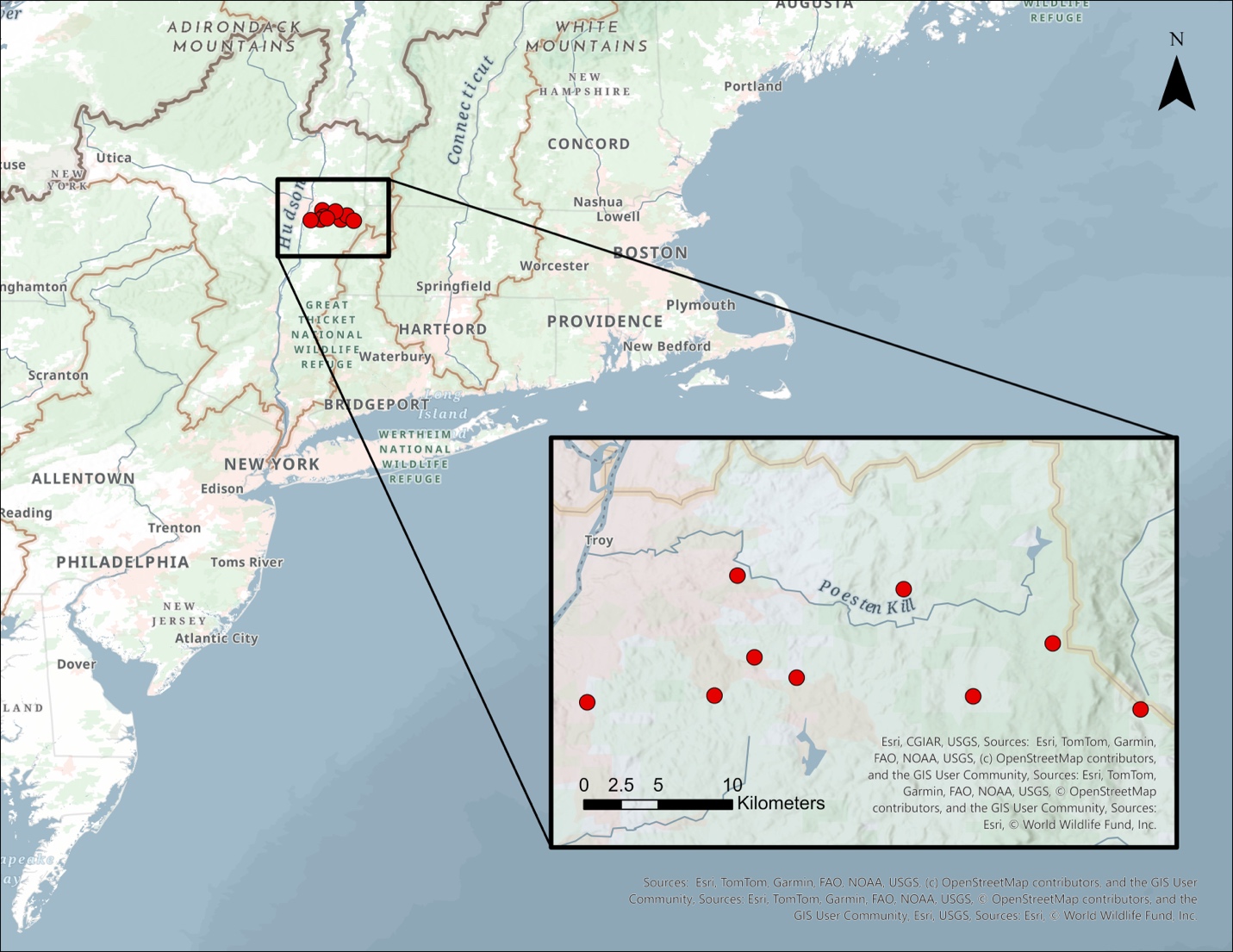

Supplement: Supplementary file 1 — Figure S1: Map of amphibian populations. [file ECE3-16-e73598-s001.docx]
